# Human T lymphocytes at tumor sites

**DOI:** 10.1007/s00281-022-00970-4

**Published:** 2022-11-16

**Authors:** Samuele Notarbartolo, Sergio Abrignani

**Affiliations:** 1 INGM, Istituto Nazionale Genetica Molecolare “Romeo Ed Enrica Invernizzi”, Milan, Italy; 2 Department of Clinical Sciences and Community Health, Università Degli Studi Di Milano, Milan, Italy

**Keywords:** Tissue-resident T cells, Trm cells, Cancer immunology, Immunotherapy, T cell homing, Memory T cell differentiation

## Abstract

CD4^+^ and CD8^+^ T lymphocytes mediate most of the adaptive immune response against tumors. Naïve T lymphocytes specific for tumor antigens are primed in lymph nodes by dendritic cells. Upon activation, antigen-specific T cells proliferate and differentiate into effector cells that migrate out of peripheral blood into tumor sites in an attempt to eliminate cancer cells. After accomplishing their function, most effector T cells die in the tissue, while a small fraction of antigen-specific T cells persist as long-lived memory cells, circulating between peripheral blood and lymphoid tissues, to generate enhanced immune responses when re-encountering the same antigen. A subset of memory T cells, called resident memory T (T_RM_) cells, stably resides in non-lymphoid peripheral tissues and may provide rapid immunity independently of T cells recruited from blood. Being adapted to the tissue microenvironment, T_RM_ cells are potentially endowed with the best features to protect against the reemergence of cancer cells. However, when tumors give clinical manifestation, it means that tumor cells have evaded immune surveillance, including that of T_RM_ cells. Here, we review the current knowledge as to how T_RM_ cells are generated during an immune response and then maintained in non-lymphoid tissues. We then focus on what is known about the role of CD4^+^ and CD8^+^ T_RM_ cells in antitumor immunity and their possible contribution to the efficacy of immunotherapy. Finally, we highlight some open questions in the field and discuss how new technologies may help in addressing them.

## Introduction

The biology of human T lymphocytes, for practical reasons, has been mostly defined with T lymphocytes purified from peripheral blood, and the study of circulating lymphocytes is still the basis of our conceptualization of T cell-mediated immunity. However, peripheral blood T lymphocytes represent only about 1–2% of the total T cell pool in the body [1]. Therefore, the great majority of T lymphocytes that are in lymphoid tissues or that infiltrate or stably reside in non-lymphoid tissues, which play pivotal roles in immune surveillance and organ homeostasis, have not been investigated thoroughly.

The existence of T lymphocytes stably residing in non-lymphoid tissues, named tissue-resident or resident memory T (T_RM_) cells, has been postulated about 20 years ago [2] and demonstrated 10 years later in mice by transplantation and parabiosis experiments [3–5]. In humans, the long-term persistence of T cells in tissues has been recently demonstrated in transplanted organs, such as lungs, intestine, and liver, in which donor T cells have been shown to reside for more than 1 year inside the transplant but have not been detected in peripheral blood of the recipients [6–10].

T_RM_ cells can be either CD4^+^ or CD8^+^ lymphocytes, dwell in non-lymphoid tissues, patrol their surroundings, and can promptly respond when re-exposed to their antigens, providing protective immunity independently of T cells newly recruited from peripheral blood [11, 12]. Upon antigen re-exposure, T_RM_ cells can trigger rapid adaptive immune responses and promote innate responses such as maturation of dendritic cells (DC) and activation of natural killer cells [13]. They can also provide bystander protection against antigenic unrelated pathogens and amplify the activation of a small number of cells to generate an organ-wide response [14].

These features enable T_RM_ cells to potentially act as critical players in the immune surveillance against solid tumors, especially at the early stages of cell transformation. In fact, the clinical manifestation of tumors implies that cancer cells have evaded immune surveillance, including that from T_RM_ cells. At this point, immune responses are mainly mediated by newly recruited effector T cells specific for tumor antigens [15] or viral antigens from infected tumor cells [16].

Compared with their protective function in infectious diseases, the precise role of T_RM_ cells in antitumor immunity is not well characterized. Nonetheless, recent studies in mouse models have provided evidence for a relevant contribution of T_RM_ cells in the immune response to different tumor types [17–20]. Moreover, several studies in human tumors have shown a positive correlation between the abundance of T_RM_ cells and a better prognosis in cancer patients (summarized in [21]).

Here, we review the current knowledge on the anatomic compartmentalization of T lymphocytes, describing how T_RM_ cells are generated during an immune response and what are the signals that guide first their migration from lymph nodes to non-lymphoid tissues and then their retention and maintenance in tissues. We then focus on what is known about the role of CD4^+^ and CD8^+^ T_RM_ cells in antitumor immune responses and their possible contribution to the efficacy of immunotherapy. Finally, we highlight some open questions in the field and discuss how new technologies, especially single-cell techniques, may help in addressing them. It is important to keep in mind that studies on T_RM_ cell function have mostly relied on investigation in animal models in vivo due to the difficulty of accessing and performing experiments on human tissue samples in vitro. That is why in this review we emphasize data on T_RM_ cell function in humans.

## Anatomic compartmentalization of T lymphocytes

**Fig. 1:**
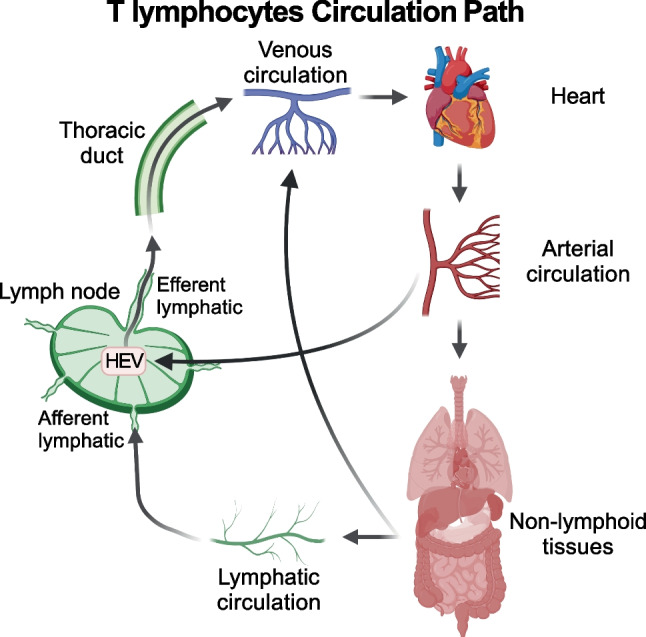
T cell trafficking in lymphoid and non-lymphoid tissues. T cells enter non-lymphoid tissues exclusively through arterial blood and exit either with lymph to reach lymph nodes via the afferent lymphatic vessels, or with venous blood. T cells can enter lymph nodes also directly from the arterial circulation through the high endothelial venules (HEV) and return to the blood circulation through the efferent lymphatic vessels and the lymph ducts

Lymphocytes patrol tissues to surveil the body against the attack of pathogens. They do so by continuously trafficking between tissues and lymph nodes. T lymphocytes can enter non-lymphoid tissues exclusively through extravasation from arteries and exit through either efferent lymphatics or veins. T cells can reach lymph nodes from efferent lymphatics or from arterial blood going into high endothelial venules; they can then return to blood circulation through efferent lymphatics merging with the lymph ducts that empty lymph into venous vessels (Fig. 1).

Different T cell populations use combinations of chemokine receptors, integrins, and selectins to migrate and home into different lymphoid and non-lymphoid tissues. For instance, the entry into secondary lymphoid organs (SLOs) depends on cell surface expression of L selectin (CD62L) and CCR7 [22, 23], while display of the selectin P ligand (CLA) guides lymphocyte migration to the skin [24], and α4β7 integrin and CCR9 lead homing to the small intestine [25, 26]. Based on the expression of CCR7, a seminal study [27] defined in human blood two populations of memory CD4^+^ and CD8^+^ T lymphocytes with distinct homing potential and effector function. On the one hand, CCR7^–^ T cells (named effector memory, T_EM_) upon activation display effector function; express receptors, such as CXCR3, CCR5, and CCR1, driving migration into inflamed tissues; and have shortened telomere length [27] suggestive of reduced self-renewal capacity of these cells. On the other hand, CCR7^+^ T cells (named central memory, T_CM_) upon activation did not display effector function, could efficiently stimulate dendritic cells, and express receptors (e.g., CD62L and CCR7) driving recirculation within blood and lymphoid tissues, but upon antigenic re-challenge, they can then differentiate toward T_EM_ cells, thus displaying effector function and expressing receptors that drive migration into inflamed tissues. Subsequent studies in mice corroborated this concept by identifying T_EM_ cells infiltrating non-lymphoid tissues [2, 28].

The expression of cell surface molecules that drive T lymphocytes toward specific tissues increases the efficiency of the immune surveillance by focusing migration of CCR7^–^ T_EM_ into sites of infection/inflammation rather than into healthy tissues [29].

Tissue-infiltrating lymphocytes differ from the circulating ones for the expression of surface markers but also for the acquisition of tissue-specific effector functions. Already 30 years ago, it was demonstrated in patients with chronic hepatitis C that intrahepatic and circulating T cell clones from the same person, specific for the same HCV protein, differed in terms of T cell receptor (TCR) sequences and in their ability to help IgA antibody production by B lymphocytes, indicating functional compartmentalization of T lymphocytes to the site of disease [30]. More recently, it has been shown by high-throughput screenings that, indeed, T lymphocytes infiltrating different human tissues are characterized by the combined expression of trafficking and functional markers that are specific to the tissue microenvironment [31, 32]. This feature adds another layer of complexity to the heterogeneity of T cell subsets from peripheral blood. The picture is further complicated by the observation that the composition of tissue-infiltrating lymphocytes varies over time during life [32, 33], possibly reflecting the history of antigenic exposure in different anatomic compartments.

These data imply that each tissue may have a “compartment code” and that T lymphocytes need to display the right password to migrate and home into that anatomic site, exert their effector function, and eventually stably reside or recirculate as memory cells to provide local or systemic long-term protection.

## Generation of T_RM_ cells

**Fig. 2:**
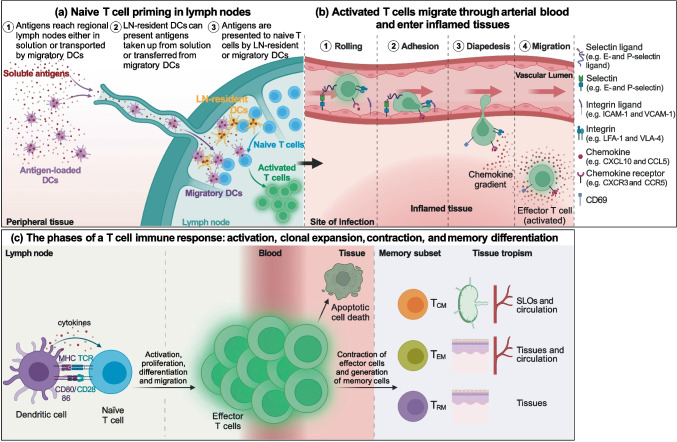
T cell activation and migration to non-lymphoid tissues. (**a**) Antigens are presented to naïve T cells either by migratory DCs, which collect antigens in the peripheral tissues, mature, and migrate into the lymph nodes, or by lymph node-resident DCs that take up antigens transported in solution with the lymph. (**b**) Activated T cells exit from lymph nodes reaching first venous and then arterial blood and home into inflamed tissue chasing a gradient of inflammatory chemokines thanks to the expression of the corresponding chemokine receptors. Once in the proximity of the inflamed tissue, T cells adhere to the vascular endothelium first through weak interactions with endothelial selectins, such as E- and P-selectin, and then arresting their rolling by binding with integrins to endothelial adhesion molecules. Stable adhesion is followed by extravasation guided by the chemokine gradient and the upregulation of CD69 expression. (**c**) Upon activation naïve T cells start to proliferate and differentiate into effector cells, which migrate to peripheral tissues where they perform their effector function. Once the antigen has been eliminated, the large majority of effector cells die by apoptosis, while a small fraction of antigen-specific T cells persists as T_CM_, T_EM_, or T_RM_ cells, which are characterized by a tropism toward different anatomic sites, to provide systemic and local long-term protection

T lymphocyte activation requires cell-to-cell contact for antigen recognition and the subsequent clonal expansion or the display of effector function. Thus, the different anatomic compartmentalization of different T cells that privilege either cell division or cytokines secretion maximizes the efficiency of target identification, elimination, and establishment of immunological memory. Indeed, naïve T cells follow a relatively restricted recirculation from blood to SLOs where they can encounter antigens drained from tissues, thus reducing the area of immune surveillance needed to mount a primary immune response. CD4^+^ and CD8^+^ naïve T lymphocytes are activated in lymph nodes (LNs) by recognizing peptide antigens presented on the major histocompatibility complex (MHC) of DCs. Antigens can be transported from the non-lymphoid tissues into the LNs by migratory DCs or arrive as soluble molecules transported with the lymph and then collected and presented to T cells by LN-resident DCs. After activation by stimulatory DCs, naïve T lymphocytes start to proliferate and differentiate into effector cells that migrate, in response to chemotactic cues, to tissues where they carry out their effector functions. Once the antigen (e.g., pathogen or cancer cell) has been cleared, most (> 90%) of effector T cells die by apoptosis, while a small fraction of antigen-specific T cells persists as T_CM_, T_EM_, or T_RM_ cells to provide systemic and local long-term memory [34, 35] (Fig. 2). Despite the extensive death of effector cells, the remaining memory T lymphocytes specific for a given antigen are numerically more represented than the initial naïve counterpart, and furthermore, they can perform faster responses and a broader immune patrolling, since, with T_EM_ and T_RM_, surveillance is extended to non-lymphoid tissues.

Differentiation of T_CM_, T_EM_, and T_RM_ cells is not clearly defined, as different models have been proposed which take into account strength and duration of TCR stimulation, and levels of inflammatory signals [36, 37]. In particular, the role of TCR stimulation strength in generating T_RM_ cells is controversial [38–42] and further complicated by the observation that TCR::pMHC affinity and the signaling strength downstream of the TCR represent independent parameters [43]. On the contrary, there is growing evidence that a T_RM_ cell fate is imprinted, at least in part, before the entry of effector T cells into peripheral tissues and possibly already during the priming of naïve T cells [44, 45]. In agreement with this hypothesis, stimulation by different DC subpopulations has been shown to instruct different tropisms of activated T cells. Mouse studies demonstrated that naïve T cell activation primarily occurs in SLOs proximal to the tissue of antigen sampling and that the tissue-emigrating DCs convey, together with the antigen, signals to polarize the migration of the activated T lymphocytes to the same non-lymphoid tissue they came from [26, 46, 47]. For instance, DCs migrating from the intestine can produce retinoic acid and TGF-β to induce the expression of the gut-homing molecules α4β7 and CCR9 on T cells [48, 49]. Similarly, cutaneous and lung DCs have been shown to instruct T cell homing to skin and lungs by inducing the expression of CCR10 and CCR4 on the T cell membrane [46, 50]. On the contrary, soluble antigens drained in lymph nodes from the afferent lymph and presented by LN-resident DCs can induce the differentiation of T_CM_-like Tcf1^+^ T cells, which are endowed with higher self-renewal capacity [51] and imprinted to recirculate within SLOs. Recently, a specific population of human migratory CD1c^+^ CD163^+^ DCs (also named DC3) has been shown to induce in vitro the generation of CD8^+^ cells with a T_RM_ signature through TGF-β signaling [52, 53]. The CD8^+^ T_RM_ cells generated were able to colonize and inhibit the progression of breast cancer in a humanized mouse model [52]. Moreover, DC3 cells were found to infiltrate human luminal breast cancer primary tumors and their frequency correlated with that of CD8^+^CD69^+^CD103^+^ T_RM_ cells [53], supporting their role as T_RM_ cell inducers. The hypothesis that the tropism of memory T cells is dictated by the nature of the stimulating antigen-presenting cells (APCs) and by the site of antigen inoculation is corroborated by the observation that some vaccination strategies can preferentially induce the generation of T_RM_ cells. For instance, the intranasal administration of a live-attenuated influenza vaccine, but not the intraperitoneal administration of an injectable inactivated influenza virus, induced the formation of virus-specific lung CD4^+^ and CD8^+^ T_RM_ cells, in mice. These virus-specific T_RM_ cells mediated protection independently from circulating T cells and could also provide heterosubtypic immunity [54].

Together these data indicate that naïve T lymphocytes can be imprinted to form a pool of T_RM_ cells depending on signals received during activation in LNs, signals that include the nature of the stimulating DCs, exposure to TGF-β, and possibly the strength and the duration of activation.

A critical step in the generation of T_RM_ cells is the recruitment of the activated T cells into the tissue. Effector T cells, differentiated from recently activated naïve T cells, enter tissues chasing chemotactic inflammatory signals released by the diseased site, such as CCL5 and CXCL9 or CXCL10 that attract activated T cells expressing CCR5 and CXCR3, respectively [55–58]. However, activated T lymphocytes can transiently acquire the ability to enter tissues in the absence of inflammation [4, 59] which can broaden the immune surveillance beyond the site of initial antigen encounter. It has been hypothesized that T_RM_ and effector T cells differentiate independently from different precursors branching early upon naïve T cell activation, since CD8^+^ T_RM_ cells have been shown to develop from tissue-infiltrating T lymphocytes that do not express the effector T cell marker KLRG1 [58, 60]. However, this hypothesis has been challenged by the finding that in vivo a sizeable fraction of KLRG1^–^ memory T cell precursors derive from effector T cells that have downregulated KLRG1 expression and that these “exKLRG1” precursors can generate T_RM_ cells [61]. The hypothesis T_RM_ cells can differentiate from effector T cells is further supported by the identification of effector T cells bearing a T_RM_-like transcriptional signature [44] and by the finding that T_RM_ cell differentiation relies on a hybrid transcriptional program regulated by both effector- and memory-specific transcription factors. While mouse T_RM_ cell differentiation is regulated by Blimp-1, its homolog Hobit, Runx3, Id2, and Id3 [19, 62, 63], there is no evidence that these transcription factors dictate the transcriptional identity of human T_RM_ cells. Although Runx3 has been reported to regulate (in cooperation with Notch signaling) the transcriptional program of both mouse and human T_RM_ cells [19, 64–66], there is no evidence of a master transcriptional regulator of human T_RM_ cells, and additional investigation is necessary to understand how the different steps of T_RM_ cell generation and maintenance are controlled.

These data indicate that T_RM_ cell precursors enter peripheral tissues as memory precursor effector cells derived from the activation of T_RM_-fate imprinted naïve T cells.

## Tissue retention and maintenance of T_RM_ cells

Once inside tissues, T_RM_ cell precursors need to receive retention signals that make them long-lived resident cells. In mice, CD69 upregulation is one of the first events that allow T lymphocytes to extravasate, enter, and persist in tissues [67]. CD69 post-transcriptionally antagonizes the sphingosine-1-phosphate receptor (S1PR1) [68], which regulates chemotaxis toward the sphingosine-1-phosphate (S1P) produced by the endothelial cells of lymph and blood vessels [69]. CD69 interacts with S1PR1 transmembrane and membrane-proximal regions and the helix 4, resulting in S1PR1 internalization and degradation without immediate effects at the transcriptional level [68]. Later, S1PR1 is also transcriptionally silenced in parallel with the downmodulation of its transcriptional regulator KLF2 [70]. Cytokines that can induce CD69 upregulation and S1PR1 downregulation include type-I interferons [71], TNF-α, and IL-33 [70]. The same mechanism of tissue retention mediated by the CD69-S1PR1 antagonism takes place in the lymph nodes where, after priming by APCs, recently stimulated T lymphocytes upregulate CD69 to transiently prolong their stay and allow full activation. For this reason, CD69 upregulation is also an early activation marker [72]. In addition to S1PR1, other sphingosine receptors can guide leukocytes toward lymph and blood vessels [73]. Among these, S1PR5 has been recently shown in the mouse to contribute to the tissue egress of antigen-experienced CD8^+^ T cells, under the transcriptional control of T-bet and Zeb2 transcription factors [74].

While CD69 expression, which limits the S1PR1 signaling and prevents T cell egress from tissues, is common to activated T cells that enter peripheral tissues, the integrins CD103 (αE integrin) and CD49a (α1 integrin) are expressed in response to TGF-β and IL-12 signaling [75, 76] and function as anchors to retain T cells within the tissue, so making them resident T cells [60, 77–79]. By binding to E-cadherin and collagen IV, respectively, CD103 (in the form of αEβ7 integrin) and CD49a (in the form of α1β1 integrin) not only anchor T_RM_ cells to the tissue but can also enhance their proliferation and limit apoptosis, thus providing survival signals [80–82]. In addition to CD103 and CD49a, also CXCR6 contributes to the localization and the maintenance of T_RM_ cells by binding to CXCL16 present on epithelial cells membrane and triggers conformational changes in integrins that support cell adhesion [83–85]. Additional molecules contributing to tissue retention of T_RM_ cells are RGS1 and RGS2 [64, 86] which accelerate the termination of heterotrimeric G protein signaling and, in turn, reduce the sensitivity to chemotactic cues [87], especially in the gut [88]. The imprinting during the priming phase may be sufficient to sensitize T_RM_ cell precursors to upregulate tissue retention molecules in response to cytokines in the tissue microenvironment, such as TGF-β and IL-12, but local re-encounter of their antigen can enhance T_RM_ cells proliferation and tissue retention [89, 90].

**Fig. 3:**
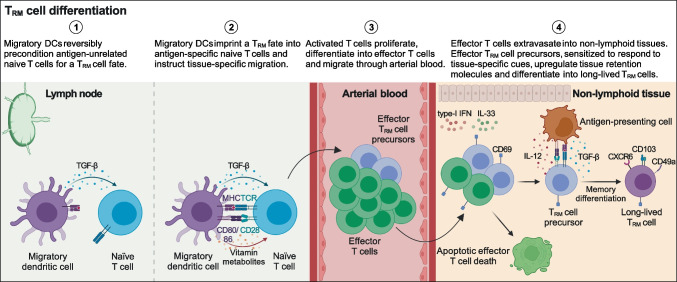
T_RM_ cell differentiation. Naïve T cells can be reversibly preconditioned in homeostasis and imprinted during activation by migratory DCs to become T_RM_ cells through TGF-β signaling. Migratory DCs can also instruct T cells to home into the same non-lymphoid tissue they came from. For instance, intestinal and cutaneous migrating DCs, by metabolizing respectively vitamin A and vitamin D, induce the expression of chemokine receptors guiding activated T cell homing into the gut and the skin. Recently activated effector T cells reach the inflamed non-lymphoid tissues through arterial circulation and extravasate also thanks to the competition between CD69 and S1PR1. Here, a subset of effector T_RM_ precursor cells upregulates the expression of tissue-retention molecules (e.g., CD103, CD49a, and CXCR6) in response to TGF-β and IL-12, differentiating into long-lived T_RM_ cells. Re-encountering the cognate antigen presented by professional and non-professional antigen-presenting cells may enhance the establishment of T_RM_ cells

In summary, data indicate naïve T cells can be preconditioned in homeostasis and imprinted during activation by migratory DCs to become T_RM_ cells through TGF-β signaling. Moreover, additional signals in tissues, such as re-encounter of their antigen and presence of cytokines such as type-I interferon, IL-33, TGF-β, and IL-12, will define whether the memory cell precursors will be retained in tissues and will differentiate into long-lived T_RM_ cells (Fig. 3).

It is worth noting that signals required to become T_RM_ cells may diverge in different tissues [91] and their systematic identification is still an open area, especially in humans.

## Markers for the identification of T_RM_ cells

The expression of CD69, CD103, and the other mentioned tissue retention molecules (Table 1) has been used in different combinations to identify T_RM_ cells. However, none of the described proteins is sufficient alone to define T_RM_ cells, nor is the contemporary expression of all of them necessary. For instance, CD69 expression alone, which has been often used in the literature for the identification of T_RM_ cells, is not sufficient to univocally define T cells residing in tissues as its upregulation represents a common feature of T lymphocytes leaving the circulation and entering peripheral tissues, including those that are not going to persist there. Such complexity in the expression patterns of surface markers may reflect differences between CD4^+^ and CD8^+^ T_RM_ cells and the presence of tissue-specific signals or the occupation of non-overlapping sub-anatomic niches [92–94]. For instance, CD4^+^ T_RM_ cells in the human dermis lack CD103 expression, whereas those in the epidermis are CD103^+^ [92]. Also, CD8^+^ CD103^+^ and CD103^–^ T_RM_ cells are found in the human intestine, where they show different preferential distribution and effector potential: both the populations infiltrate the intestinal epithelium and lamina propria, but the CD103^+^ T_RM_ cells are more frequent in the epithelium and express higher IL-2 and IL-7Rα, while CD103^–^ T_RM_ cells are more frequent in the lamina propria and produce higher granzyme K [95].

**Table 1:** Expression and function of molecules associated with T_RM_ cells

Subcellular localization	Name	Alias	Gene name	Expression in T_RM_ cells	Function
Cell membrane					
	CD69	AIM, CLEC2C	*CD69*	Upregulated	Post-transcriptionally antagonizes S1PR1 and prevents tissue egress
	Sphingosine 1-phosphate receptor 1 (S1PR1)	S1P1, CD363	*S1PR1*	Downregulated	Regulate chemotaxis toward S1P, guiding the migration to lymph and blood vessels
	Sphingosine 1-phosphate receptor 5 (S1PR5)	S1P5, EDG8	*S1PR5*	Downregulated	
	Integrin alpha-E (CD103)	αE integrin	*ITGAE*	Upregulated	Binds E-cadherin and conveys survival signals; provides co-stimulation and polarizes the exocytosis of lytic granules
	Integrin alpha-1 (CD49a)	α1 integrin, VLA1	*ITGA1*	Upregulated	Binds collagen IV and provides survival signals
	C-X-C chemokine receptor type 6 (CXCR6)	CD186, BONZO	*CXCR6*	Upregulated	Binds CXCL16, also in its membrane-bound form; triggers conformational changes in integrins to support cell adhesion
Intracellular					
	Krueppel-like factor 2 (KLF2)	LKLF	*KLF2*	Downregulated	Transcriptional activation of S1PR1 and CD62L; transcriptional repression of CCR5 and CXCR3
	Regulator of G-protein signaling 1 (RGS1)	BL34, IER1	*RGS1*	Upregulated	Terminate heterotrimeric G protein signaling and reduce cell sensitivity to chemotactic cues
	Regulator of G-protein signaling 2 (RGS2)	G0S8	*RGS2*	Upregulated	

Here, we propose to define T_RM_ cells based on the expression of at least one of the molecules mediating tissue retention, i.e., CD103, CD49a, and CXCR6, and on the simultaneous lack of molecules, such as S1PR1 and S1PR5, which guide T cell egress from tissues (Table 2). This combination of markers cannot inform on the long-term tissue retention of T_RM_ cells but provides a snapshot of the cell status at a defined time point without excluding the potential of leaving non-lymphoid tissues under different circumstances. In any case, there is evidence that T_RM_ cells re-entering the circulation upon reactivation are biased to return to the tissue of origin and become again resident T cells [96].

**Table 2:** Possible combinations of cell-surface molecules defining T_RM_ cells

Function	Surface marker	Combinations of markers defining T_RM_ cells						
Tissue-retention	CD103	+	+	+	–	+	–	–
	CD49a	+	+	–	+	–	+	–
	CXCR6	+	–	+	+	–	–	+
Recirculation	S1PR1	–	–	–	–	–	–	–
	S1PR5	–	–	–	–	–	–	–

The expression of at least one of the molecules mediating tissue retention and the simultaneous lack of receptors guiding T cell egress from tissues is required to define T_RM_ cells. “ + ”, expression; “–”, lack of expression.

As for the identification of tissue-specific signals driving T_RM_ cell differentiation, a comprehensive definition of cell markers that identify CD4^+^ and CD8^+^ T_RM_ cells at different anatomic and sub-anatomic sites is still missing.

## T_RM_ cells in tumor immunity

**Fig. 4:**
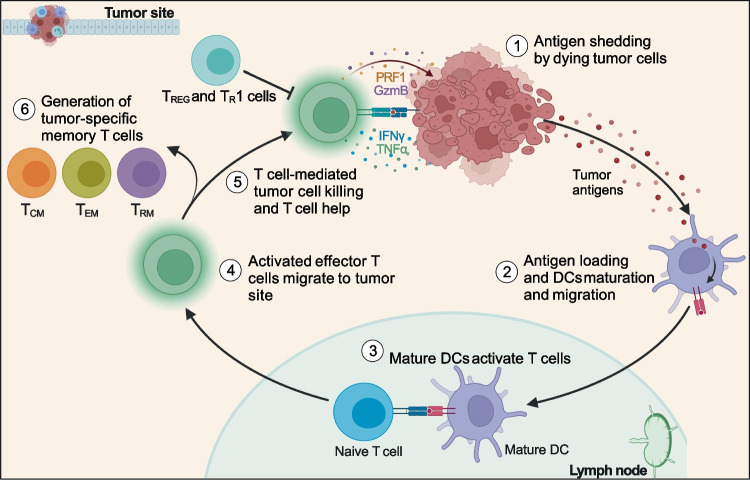
Generation of tumor-specific effector and memory T cells. Tumor antigens released by cancer cells are collected by dendritic cells and presented to naïve T cells in lymph nodes. Differentiated tumor-specific effector T cells migrate to the tumor site where they perform their effector function. Effector CD8^+^ and CD4^+^ T cells exert their anti-tumor activity by directly killing tumor cells and providing help to other immune cells, while T_REG_ and T_R_1 cells suppress the immune response. Immune-mediated cancer cell death can induce the release of more tumor antigens and sustain anti-tumor immunity

**Fig. 5:**
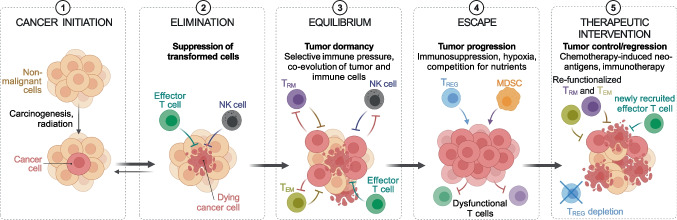
Tumor and immune system co-evolution. Tumor-specific T_RM_ cells can be generated following the effector response against the first immunogenic cancer cells. During the equilibrium phase of tumor development, T_RM_ cells can kill cancer cells and contribute to the control of tumor growth. However, the tumor can escape immune surveillance, including that from T_RM_ cells, and generate an immunosuppressive microenvironment. Chemotherapy and immunotherapy can reactivate the anti-tumor immune response: chemotherapy by inducing the release of tumor neoantigens, which can trigger the generation of new tumor-specific effector T cells; immunotherapy by reactivating the effector function of dysfunctional memory T cells

Accumulating evidence shows that T_RM_ cells frequently reside in human tumors, especially those of epithelial origin, and can have a protective function [21, 97, 98]. How T_RM_ cells participate in controlling tumor growth has not been determined yet, nor is the contribution of tumor-specific or bystander T_RM_ cells clear. The pool of intra-tumor T_RM_ cells can be made of lymphocytes present in the tissue before the appearance of transformed cells, such as those elicited by microbes and not specific for tumor antigens, and of T cells specific for tumor-derived antigens. Tumor-specific T_RM_ cells can be generated following the effector response against immunogenic transformed cells (Fig. 4). During the equilibrium phase of tumor development, they can kill transformed cells and contribute to the control of tumor growth [99] (Fig. 5). However, clinical manifestation of tumors implies that cancer cells have evaded immune surveillance, for instance by inducing T cell dysfunction. Dysfunctional T_RM_ cells can be reactivated by blockade of inhibitory checkpoints (e.g., CTLA4 and PD1) and they can contribute to the efficacy of immunotherapy (see below). Nevertheless, de novo immune responses triggered by tumor neoantigens made available by radio- or chemotherapy-induced immunogenic cell death [100] are usually mediated by newly generated effector T cells (Fig. 5). In this context, new tumor-specific activated T cells can migrate from LNs in blood circulation and then into cancer tissue where they can exert effector function [15]. Consistently with this scenario, we observed in CRC patients treated with neoadjuvant chemotherapy that the T cells that infiltrate CRC liver metastasis and show the highest clonal expansion are effector T cells. These effector T cells show evidence of TCR-mediated activation and lack typical features of T_RM_ cells (S.N. and S.A, unpublished), suggesting they have been recently recruited from circulation. Longitudinal studies would be required to assess whether such effector responses are able to generate memory T cells and if these cells could become resident due to tumor microenvironmental factors, such as TGF-β.

### CD8^+^ T_RM_ cells

The abundance of tumor-infiltrating CD8^+^ CD103^+^ T cells and the elevated expression of a T_RM_ signature correlate significantly with prolonged disease-free or overall survival of patients with many different tumor types, including breast cancer [101, 102], lung cancer [17, 103–105], colorectal cancer (CRC) [106], melanoma [15, 107, 108], head and neck cancer [109], esophageal cancer [110], gastric cancer [111], hepatocellular carcinoma [112], ovarian cancer [113–115], cervical cancer [116], endometrial adenocarcinoma [117], and bladder cancer [118]. Also, the localization of T_RM_ cells within the tumor matters. Indeed, the infiltration of CD103^+^ T cells in the tumor epithelium, but not in the tumor stroma associates with longer survival, as reported in breast [101, 102] and lung [104] cancers. The prognostic power associated with higher T_RM_ cell abundance might vary depending on the tumor stage and is higher in early-stage disease when T_RM_ cells are probably still functional.

The abundance of CD8^+^ CD103^+^ T cells has been negatively correlated with the presence of metastasis in patients with gastric adenocarcinoma [111], but the role of T_RM_ cells in protecting from cancer spreading has to be clarified yet. Nonetheless, recent evidence from a mouse model of melanoma demonstrated that T_RM_ cell activation can trigger the maturation and migration of dermal DCs to LNs, and induce new cytotoxic CD8^+^ T cell responses that suppress the growth of the primary tumor and of melanoma cells disseminated to the lung [15].

Tumor-specific CD8^+^ T_RM_ cells can directly kill tumor cells in a MHC-I-restricted manner [109] through the production of cytotoxic molecules. Indeed, they can express high levels of granzyme B, perforin, and the degranulation marker LAMP-1 (CD107a) [103, 105, 119]. Notably, T_RM_ cells in ovarian cancer show features of activated T cells, such as an elevated surface expression of HLA-DR coupled to the downregulation of CD127 (IL-7Rα), and the co-expression of the proliferation marker Ki67 [113]. Both CD103^+^ and CD49a^+^ CD8^+^ T_RM_ lymphocytes are potent killer cells [107]. CD103 is recruited at the immunological synapse and provides co-stimulatory signals [120]. Its interaction with E-cadherin on target cells promotes the phosphorylation of ERK1/2 and phospholipase Cγ1 in T_RM_ cells and is required to polarize the exocytosis of cytolytic granules and optimize the killing of the target [119, 120]. However, CD49a^+^ T_RM_ cells have been reported to have a superior protective function than CD103^+^ ones, at least in a mouse model of airway viral infection, possibly due to their relatively higher local motility that allows a more efficient patrolling of the tissue [121].

At the same time, tumor-specific T_RM_ cells can promote antitumor immunity through the release of cytokines and chemokines that help activation or recruitment of other immune cells [15]. Indeed, in mice, effector cytokines released by T_RM_ cells can stimulate local DCs, natural killer cells, and other T cells [13, 122, 123].

T_RM_ cell effector function also depends on the nature of the stimulating APC. In principle, CD8^+^ T_RM_ cells can be activated by APCs of non-hematopoietic origin, including tumor cells, or by APCs of hematopoietic origin through antigen cross-presentation. In a mouse model of viral infection, lung CD8^+^ T_RM_ cell activation by professional or non-professional APCs resulted in different functional outputs: antigen presentation by non-hematopoietic APC induced T_RM_ cells proliferation and the expression of proinflammatory cytokines, likely to eliminate infected cells; on the contrary, antigen presentation by hematopoietic APCs elicited T_RM_ cells to express several interferon-responsive genes but limited the production of some pro-inflammatory cytokines and chemokines, such as *Ccl3*, *Ccl4*, *Ifng*, and *Xcl1*, possibly to prevent excessive local inflammation and leukocytes recruitment [124].

Collectively, the described features indicate CD8^+^ T_RM_ cells can display anti-tumor effector responses, but additional investigation is still needed to dissect the role of CD8^+^ T_RM_ cell subsets expressing different tissue retention molecules, such as CD103, CD49a, and CXCR6.

### CD4^+^ T_RM_ cells

Although CD4^+^ T_RM_ lymphocytes that accumulate in tissues often outnumber CD8^+^ T_RM_ cells [125], most studies on T_RM_ cells have focused on CD8^+^ T cells and the understanding of CD4^+^ T_RM_ cell biology is less advanced. Tissue-resident CD4^+^ T_H_1, T_H_2, T_H_17, and regulatory T cells (T_REG_) have been identified in many tissues where they have been shown to protect against pathogens but also to exacerbate inflammatory and autoimmune diseases [126–129]. Instead, the functional dissection of CD4^+^ T_RM_ cells has been complicated by their heterogeneity [31].

The knowledge gap of CD4^+^ T_RM_ cells is even bigger in the context of anti-tumor immunity. In the last decade, growing evidence has indicated that CD4^+^ T cells can contribute to control tumor growth in humans [130–134], and CD4^+^ T cells specific for neo-antigens have been detected within tumors with elevated Tumor Mutational Burden, such as melanoma [132]. CD4^+^ T cells are necessary for the priming and differentiation of CD8^+^ T cells [135] and support durable tumor-specific cytotoxic T cell responses by instructing the downregulation of coinhibitory receptors and enhancing the capacity of CD8^+^ T cells to infiltrate tumors [136]. Moreover, CD4^+^ T cells can cooperate with cytotoxic T lymphocytes in bystander killing of cancer cells [137] and can also acquire cytotoxic function so as to kill tumor cells expressing MHC-II as effectively as CD8^+^ T cells [138, 139]. In addition, CD4^+^ T cells with a T_FH_-like phenotype (CXCR5^+^/PD-1^+^/CXCL13^+^) colocalize with B lymphocytes in tertiary lymphoid structures (TLSs) that are particularly present within non-epithelial tumors, such as melanoma and sarcoma, and the presence of these TLSs correlates with better responses to immunotherapy and longer patients survival [140, 141].

T_H_17 cells have been reported to infiltrate human ovarian and colon cancer [142, 143]. Tumor-infiltrating T_H_17 cells produced cytokines, such as CXCL9 and CXCL10, contributing to the recruitment of effector T cells. In the context of ovarian cancer, the abundance of T_H_17 cells correlated with patient survival and was reduced in more advanced stages [143]. Although in this study tumor-infiltrating T_H_17 cells were not tested for the expression of T_RM_ cell markers, they were shown to express several integrins and to be long-lived. Moreover, their resistance to apoptosis, mediated by Bcl-2 expression, depended on Hif-1α and Notch [142], two transcription factors involved in the regulation of human T_RM_ cell phenotype. Notably, IL-17-producing CD4^+^ T_RM_ cells have been found in the lung of patients infected with *Mycobacterium tuberculosis* and SARS-CoV-2 [129, 144], and in transplanted intestines [95]. Moreover, fate-mapping experiments using an IL-17A-reporter mouse model, combined with the analysis of clonal distribution by TCR sequencing, demonstrated that a significant fraction of lung CD4^+^ T_RM_ cells, including IL-17-negative ones (exT_H_17 T_RM_ cells), derive from effector T_H_17 cells upon *Klebsiella pneumoniae* infection [145]. These data suggest that T_H_17 cells may represent one of the major CD4^+^ T_RM_ cell populations at mucosal sites, possibly guided in their homing by the chemokine receptor CCR6. Supporting this hypothesis, a subset of T_H_17 cells expressing upon activation the immunoregulatory cytokine IL-10 and tissue residency-associated molecules, such as *CD69*, *CXCR6*, and *ITGB7*, has been identified in the circulation of human subjects, possibly representing T_RM_ cell precursors [146].

More recently, CD4^+^ CD103^+^ T_RM_ cells infiltrating human non-small cell lung cancer (NSCLC) were shown to produce IFN-γ and TNF-α, resembling a T_H_1 phenotype. The authors suggested that the IFN-γ produced by CD4^+^ T_RM_ cells could contribute to attracting CD8^+^ T_RM_ cell precursors and showed that the frequency of the two populations infiltrating NSCLC positively correlated [147].

T_REG_ lymphocytes abundantly infiltrate CRC, NSCLC, and breast cancer and have a distinct profile from circulating T_REG_ cells [148, 149]. The abundance of tumor-infiltrating T_REG_ (TI-T_REG_) cells is associated with a poor prognosis in human cancer. Although TI-T_REG_ cells do not show enhanced expression of T_RM_ cell markers compared to other tumor-infiltrating T cell populations, they constitutively express high levels of CCR8 [148, 149], which can mediate tissue retention in epithelial tissues and has been reported as a feature of T_RM_ cells in human skin [150].

Another population of suppressive CD4^+^ T cells, characterized by the lack of FOXP3 and increased GZMK expression, named T_R_1 cells, has been identified to infiltrate different human solid tumors. Based on single-cell TCR sequencing analysis, T_R_1 cells are clonally unrelated to FOXP3^+^ T_REG_ cells and clonally expanded in tumors. Their abundance, measured by the expression of the specific marker *CHI3L2*, negatively correlates with patients’ survival but positively correlates with the response to immunotherapy [151]. Despite tumor-infiltrating T_R_1 cells were not specifically tested for the expression of genes characteristic of T_RM_ cells, they express CCR5 [152], which is required for the migration toward inflamed tissues and can contribute, in combination with CCL5 locally produced by macrophages, to CD4^+^ T_RM_ cells tissue retention [55, 153].

In conclusion, more investigative efforts are needed to dissect the phenotypic and functional heterogeneity of CD4^+^ T_RM_ cells and to better understand their role in anti-tumor immunity.

## T_RM_ cells in immunotherapy

The development of single-cell sequencing technologies coupled with TCR sequencing has provided a new powerful tool to deeper investigate the heterogeneity of tumor-infiltrating lymphocytes. Indeed, TCR represents the identity card of T lymphocytes and knowledge of its sequence allows to track individual T cell clones in different tissues. Several studies have investigated at the single-cell resolution the transcriptional identity, and sometimes clonal distribution, of T cells infiltrating human tumors, including breast cancer [154, 155], NSCLC [156], CRC [157], hepatocellular carcinoma [158], bladder cancer [159], head and neck cancer [160], and basal cell carcinoma [161, 162]. However, only some of these studies have taken into consideration phenotypes specifically associated with tumor-infiltrating T_RM_ cells. The most relevant feature of these T_RM_ cells, identified as CD8^+^ CD103^+^ T cells, common to breast, lung, and basal cell cancers, was an elevated expression of genes coding for immune checkpoint receptors, such as *PDCD1* (PD-1), *CTLA4*, *HAVCR2* (TIM-3), *TIGIT*, and *LAG3*, and a higher expression of *GZMB* (granzyme B) and *PRF1* (perforin) compared with CD8^+^ CD103^–^ T cells [155, 156, 161, 163]. A subset of the CD8^+^ T_RM_ cell population also expressed genes associated with cell proliferation. Moreover, in lung cancer, a CD8^+^ CD103^+^ T cell subset with low PD-1 expression has been also identified and proposed to represent a “pre-exhaustion” state [156]. The expression of immune checkpoint receptors by tumor-infiltrating human CD8^+^ T_RM_ cells has been confirmed at the protein level by several independent studies [17, 103, 105, 113, 117, 164].

The presence of proliferation markers and the identification of T_RM_ cell subsets with a graded expression of immune checkpoint receptors suggests that T_RM_ cells may include tumor-specific T cells that have become dysfunctional upon chronic stimulation in the tumor microenvironment. Interestingly, immunotherapy with anti-PD-1 boosts the formation of T_RM_ cells in an adoptive cell transfer protocol in melanoma-bearing mice [20]. In humans, the number of infiltrating CD8^+^ CD103^+^ T_RM_ cells significantly increases in melanoma patients early upon PD-1 blockade, especially in patients responding to immunotherapy [108]. Similarly, in NSCLC patients, the density of CD8^+^ CD103^+^ T_RM_ cells before immunotherapy positively associates with improved outcomes and increases during the therapy in most of the responders, but not in non-responder patients [165]. Also, in esophageal squamous cell carcinoma, CD8^+^ CD103^+^ T_RM_ cells show enhanced proliferation and cytotoxic potential, measured by Ki67 and CD107a staining, compared to tumor-infiltrating CD8^+^ CD103^–^ cells after anti-PD-1 therapy [110]. Notably, PD-1 blockade on CD8^+^ T_RM_ cells, freshly isolated from human lung cancer, promoted a strong MHC-I-restricted cytolytic activity against autologous tumor cells ex vivo, which was impaired by blocking CD103 interaction with target cells [103]. These data indicate that immunotherapy unleashes the effector capacity of T_RM_ cells and possibly induces their proliferation. Whether the increase in T_RM_ cell infiltration upon PD-1 blockade is also sustained by the generation of new T_RM_ cells remains to be addressed.

The expression of immune checkpoint receptors is present also in T_RM_ cells from normal tissues [58], but the fact that tumor-infiltrating T_RM_ cells are less capable to produce effector molecules suggests the acquisition of a deeper state of dysfunction [166], which may be one of the reasons why cancer develop despite the presence of tumor-specific T_RM_ cells. Altogether, data from the literature indicate that CD8^+^ CD103^+^ T_RM_ cells include tumor-specific exhausted T lymphocytes that can be revitalized by immunotherapy. Therefore, inducing a pool of tumor-specific T_RM_ cells, for instance by specific vaccination strategies [17], combined with immunotherapy may represent a possible therapeutic strategy to promote tumor regression.

## T_RM_ cell maintenance in the tumor microenvironment

Considering that TGF-β can be highly expressed in the tumor microenvironment and that it regulates T_RM_ cell formation and maintenance [167, 168], it is not surprising that tumors are frequently populated by T_RM_ cells. Also, hypoxia, another environmental cue characterizing the tumor microenvironment has been recently shown to synergize with TGF-β to induce a T_RM_ phenotype on in vitro stimulated human CD8^+^ T cells [169].

Differently from effector T cells that mainly rely on glucose and glutamine metabolism [170], T_RM_ cells express high levels of the free-fatty acid (FA) transporters Fabp4 and Fabp5 and catabolize short-chain free-FA as a preferential energy source [171]. Relying on FA metabolism could give an advantage to T_RM_ cells by limiting the competition for nutrients with tumor cells, which usually consume glucose and create local hypoglycemia [172]. Indeed, promoting FA metabolism with a PPAR-α agonist sustains CD8^+^ T cell effector function, delays tumor progression, and enhances the therapeutic efficacy of PD-1 blockade in a mouse melanoma model [173]. In turn, immune checkpoint blockade promotes Fabp4 and Fabp5 expression in T_RM_ cells, but not in tumor cells, promoting lipid uptake and improving their survival [111]. Moreover, T_RM_ cells from melanoma and gastric adenocarcinoma patients show an increased FA uptake compared to the CD103^–^ infiltrating and the circulating T lymphocytes [111, 173]. However, since FA oxidation requires oxygen consumption, the lipid metabolism under hypoxic conditions usually leads to lipogenesis, making the lipid storage an inaccessible energetic source until reoxygenation. It is possible that, differently from acute hypoxia, in chronic limiting oxygen conditions, cells may reset their metabolism and prioritize the oxidation of FA and glutamine over pyruvate using the spare oxygen available [174]. Nonetheless, it remains to be addressed if and how T cells in the tumor microenvironment metabolized intracellular lipid storages for energy generation or if T_RM_ cells can adapt their metabolism to exploit other nutrients available in different tumor niches.

Targeting metabolism to boost T cell anti-tumor activity is a field of active investigation. For instance, T lymphocytes with increased intracellular L-arginine levels display enhanced survival and anti-tumor effector capacity [175], and tumor colonization with an *Escherichia coli* strain engineered to continuously convert ammonia to L-arginine increases the number of tumor-infiltrating T cells and synergizes with PD-L1 blockade in the clearance of tumors, in mice [176]. Together these data indicate that manipulation of T cell (including T_RM_ cell) metabolism represents a new area of immunotherapy.

## Conclusions

T_RM_ cells constantly patrol tissues and their adaptation to the local microenvironment makes them professional tissue defenders. Current knowledge from mouse and human studies supports a role for T_RM_ cells in anti-tumor immunity, especially in the first stages of tumor development, and growing evidence suggests they may enhance the efficacy of immunotherapy, highlighting their possible contribution to controlling tumor growth also after its clinical manifestation.

So far, analyses of T_RM_ cells in human tumors have mostly relied on the expression of one (e.g., CD103) or a few markers, but it is evident that T_RM_ cells expressing the combination of different molecules may represent subsets occupying different anatomic niches and having different patrolling and effector capacity. Moreover, research efforts have largely focused on CD8^+^ T_RM_ cells, while our understanding of CD4^+^ T_RM_ cell function is still very limited. The anti-tumor activity of T_RM_ cells may change depending on the tissue of origin and on tumor mutational load, and the role of T_RM_ cells in protecting from the metastatic spread remains unclear. Thus, we need to systematically dissect the function of the different CD4^+^ and CD8^+^ T_RM_ cell subsets from different tissues and from various primary and metastatic tumors.

The development of high-throughput technologies, including multiparametric flow and mass cytometry, microscopic tissue spatial analysis, and single-cell RNA and TCR sequencing, will certainly help in gaining novel insights into the biology of human T_RM_ cells isolated from clinical material. For instance, combining the information from single-cell RNA and TCR sequencing allows for dissecting the phenotype and the clonal expansion of T cells specifically infiltrating tumor tissues and the identification of expanded T cell clones, which may include tumor-specific T cells. Knowing the proportion of intratumor-expanded T_RM_ cells compared to other infiltrating T cell populations may reveal the relative contribution of circulating and resident T lymphocytes in anti-tumor immunity, while the dissection of their transcriptional profile may inform about the contribution of T cell subsets with different identities and the activation or silencing of specific signaling pathways. However, without prior knowledge of the tumor antigens, we are still not able to distinguish in this kind of analysis which are, among the expanded T cell clones, the tumor-specific T cells. Although the expression of CD39 has been proposed to identify tumor-specific T lymphocytes [109, 177], its expression may simply tag chronically stimulated T cells that become exhausted. These cells are likely to be enriched in tumor-specific T cell clones in the tumor microenvironment, but CD39^–^ tumor-specific T cells have been also identified and shown to have a higher proliferation capacity upon re-challenge compared to the CD39^+^ T cells [178]. Thus, the identification of better markers associated with tumor-specific T cells would be extremely valuable.

In addition to immunotherapy, T_RM_ cells could be targeted to enhance the efficacy of other immune interventions, such as vaccination or adoptive cell transfer. Indeed, in both settings, the induction of T_RM_ cells enhanced their efficacy, at least in mice [17, 20]. Therefore, improving our understanding of the biology of T_RM_ cells, including how they are generated and maintained in different human tissues, to what extent they participate in immune responses both in health (vaccination) and in disease (anti-tumor or anti-pathogen responses), and why they become dysfunctional in the tumor microenvironment, will contribute to the development of novel targeted immunotherapies.
